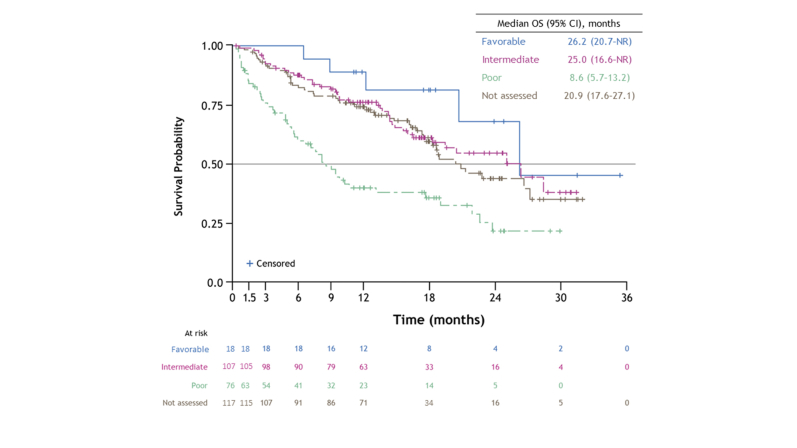# Nivolumab in patients with advanced renal cell carcinoma in France: interim results of the observational, real-world WITNESS study

**DOI:** 10.1016/j.esmoop.2024.103602

**Published:** 2024-06-18

**Authors:** P. Barthélémy, L. Albigès, B. Escudier, B. Narciso, P. Bigot, M. Chehimi, S. Emambux, F. Calcagno, G. Mouillet, J.-C. Eymard, F. Schlürmann, S. Bailly, D. Garbay, J.-F. Berdah, M.B. Palmaro, M.G. Goupil, D. Spaeth, S. Néré, C. Quentric, Y.-A. Vano, A. Thiery-Vuillemin

**Affiliations:** 1 Department of Medical Oncology, Institut de Cancérologie Strasbourg Europe, Strasbourg; 2 Department of Medical Oncology, Gustave Roussy Cancer Campus, Paris; 3 Department of Medical Oncology, Centre Hospitalier Universitaire de Tours, Tours; 4 Department of Urology, Centre Hospitalier Universitaire d’Angers, Angers; 5 Department of Medical Oncology and Hematology, Centre Hospitalier de Saint-Quentin, Saint-Quentin; 6 Department of Medical Oncology, Centre Hospitalier Universitaire de Poitiers, Poitiers; 7 Department of Medical Oncology, Centre Hospitalier Universitaire de Besançon, Besançon; 8 Department of Medical Oncology, Institut Jean Godinot, Reims CEDEX; 9 Department of Medical Oncology, Centre Hospitalier Intercommunal Quimper, Quimper; 10 Department of Thoracic Oncology, Centre Hospitalier Universitaire de Clermont-Ferrand, Clermont-Ferrand; 11 Department of Medical Oncology, Clinique Tivoli-Ducos, Bordeaux; 12 Department of Medical Oncology and Radiotherapy, Hôpital privé Toulon Hyères—Sainte Marguerite, Hyères; 13 Department of Medical Oncology, Hôpital Nord, Marseille; 14 Department of Medical Oncology, Centre Hospitalier Universitaire de Bordeaux, Hôpital Saint-André, Bordeaux; 15 Department of Medical Oncology, Polyclinique de Gentilly, Nancy; 16 Department of Medical Affairs, Bristol Myers Squibb, Paris; 17 Department of Medical Oncology, Hôpital Européen Georges Pompidou, APHP Centre—Université de Paris, Paris, France

**Keywords:** advanced renal cell carcinoma, effectiveness, metastases, nivolumab, real-world data, safety

## Abstract

**Background:**

Nivolumab is the first immune checkpoint inhibitor approved in Europe for the treatment of advanced renal cell carcinoma (aRCC) in patients resistant to prior antiangiogenic therapy. WITNESS is an ongoing, prospective, observational study designed to evaluate the effectiveness and safety of nivolumab in patients with aRCC treated in real life (or routine practice) in France (ClinicalTrials.gov identifier: NCT03455452).

**Patients and methods:**

This study includes adult patients with a confirmed diagnosis of aRCC who have initiated nivolumab after 1-2 prior lines of antiangiogenic therapy. Endpoints include overall survival (OS), progression-free survival (PFS), duration of treatment (DOT), duration of response (DOR), overall response rate (ORR), subgroup analyses, and treatment-related adverse events (TRAEs). Results after a median follow-up of 12.3 months are presented here.

**Results:**

A total of 325 patients with aRCC were included, of whom 38.2% had a Karnofsky score <80, 77.8% received nivolumab as second-line therapy, and 69.5% had undergone a previous nephrectomy. In the overall population, median OS was 20.5 [95% confidence interval (CI) 17.6-25.0] months and median PFS was 5.2 (95% CI 4.5-5.9) months. ORR was 34.5%, median DOT was 3.8 months, and median DOR was 16.5 months. Nivolumab was effective in different subgroups including patients with bone or glandular metastases and those receiving baseline corticosteroids. Moreover, effectiveness was observed irrespective of prior nephrectomy and line of treatment. No new safety signals were identified; TRAEs of any grade were reported in 32.0% of patients, grade ≥3 and serious TRAEs in 11.1% each, and TRAEs leading to discontinuation in 8.9%.

**Conclusions:**

Preliminary results of the ongoing WITNESS study confirm the real-world effectiveness and safety of nivolumab monotherapy in previously treated patients with aRCC. Treatment benefits were similar to those observed in the pivotal phase III CheckMate 025 randomized clinical trial, despite a broader, real-life study population.

## Highlights

• WITNESS is a prospective, non-interventional study of nivolumab monotherapy in patients with aRCC in France.
• Patients with a broad range of demographic/pretreatment features were included, in contrast with randomized clinical trials.
• Nivolumab monotherapy effectiveness and safety in the overall population were aligned with the phase III CheckMate 025 study.
• Nivolumab was effective in patients regardless of baseline comedications and also irrespective of the line of treatment.
• Safety results were consistent with the known safety profile of nivolumab, with no new safety signals identified.

## Introduction

According to the Global Cancer Observatory report, kidney cancer was the sixth most common cancer in France, with 15 908 new cases, a 5-year prevalence rate of 66.2 per 100 000 individuals, and 5300 deaths in 2018.^1^ Renal cell carcinoma (RCC) is the most common type of kidney cancer globally and accounts for >90% of cases.^2^ At diagnosis, ∼25% of patients have advanced RCC (aRCC; i.e. stage III or IV tumors),^3^ and advanced or metastatic disease occurs following resection with curative intent in ∼20%-30% of patients who are diagnosed at an earlier stage.^4^^,^^5^

Nivolumab, a fully human monoclonal antibody targeting the programmed cell death protein 1 (PD-1) receptor, was the first immune checkpoint inhibitor approved in Europe for the treatment of aRCC in patients resistant to prior antiangiogenic therapy.^6^ Nivolumab selectively blocks the interaction between PD-1, expressed on activated T cells, and programmed death-ligands 1 and 2 (PD-L1/L2), expressed on immune cells and tumor cells.^6^

Regulatory approval of nivolumab was based on its clinical efficacy and manageable safety profile demonstrated in CheckMate 025, a pivotal randomized phase III clinical trial of patients with previously treated aRCC that compared nivolumab (3 mg/kg every 2 weeks) with everolimus (10 mg once a day) until progression or unacceptable toxicity.^7^ Efficacy of nivolumab was demonstrated with regard to the primary endpoint of overall survival (OS), and the secondary endpoint [overall response rate (ORR)],^7^ with durable responses observed over a minimum of 5 years of follow-up.^8^

Since the strict selection criteria in randomized clinical trials only partially reflect real-life patients, studies of large real-world populations can provide valuable data regarding the effectiveness and safety of medicines in routine practice that can guide therapeutic decisions.^9^ WITNESS is an ongoing, prospective, observational, real-world study of nivolumab monotherapy as a second- or third-line treatment (cohort 1) or nivolumab plus ipilimumab as a first-line treatment (cohort 2) in patients with aRCC. This report focuses on interim results from the nivolumab monotherapy cohort (cohort 1) and aims to describe the clinical characteristics and demographics of patients with aRCC who receive nivolumab monotherapy in routine practice in France. Furthermore, we have assessed the effectiveness and safety of nivolumab in this patient population and by subgroups of interest.

## Patients and methods

### Study design

The WITNESS study is being conducted at 62 centers throughout France (ClinicalTrials.gov identifier: NCT03455452). Patients in cohort 1 are receiving nivolumab monotherapy as second- or third-line treatment according to the French marketing authorization. The study plans to follow each patient for 3 years from the index date (i.e. treatment initiation) until death, withdrawal of consent, loss of follow-up/record, or to the end of study, whichever comes first. During the follow-up period, assessments are carried out according to routine local clinical practice. Data entry in the electronic case report form is scheduled to take place at day 0, week 6, and months 3, 6, 9, 12, 18, 24, 30, and 36.

The study was approved by the French Ethics Committee, regulatory authorities, and/or other local governance bodies, and is being conducted in compliance with the International Society for Pharmacoepidemiology Guidelines for Good Pharmacoepidemiology Practices and the European Union’s Guidelines on Pharmacovigilance for Medicinal Products for Human Use. Informed oral consent was obtained from all patients in accordance with French Health Authority guidelines before any study procedures were being undertaken.

### Patient population

The study includes patients aged ≥18 years with a confirmed diagnosis of aRCC who have received 1-2 prior lines of therapy and are starting nivolumab for the first time. Key exclusion criteria are malignancies other than aRCC within 5 years before enrollment, previous treatment with anti-PD-1, anti-PD-L1, or anti-cytotoxic T-lymphocyte-associated protein 4 therapy, current involvement in an interventional clinical study, or pregnancy.

### Study endpoints

The primary endpoint is OS (defined as the time from the first administration of nivolumab to death from any cause) estimated over a 3-year follow-up period and will be reported separately when survival data reach maturity. OS is estimated based on a minimum 14-month and median 24.7-month follow-up for the current interim analysis. Secondary endpoints include estimated OS according to subgroups of interest; duration of treatment (DOT) and median duration of response (DOR) with nivolumab; progression-free survival (PFS; defined as the time from the first administration of nivolumab to the first disease progression or last known tumor assessment date, or death due to any cause, whichever occurs first); best overall response (BOR; defined as the highest level of response to nivolumab therapy); investigator-assessed ORR [defined as the proportion of patients with complete response (CR) or partial response (PR)]; disease control rate [DCR; defined as the proportion of patients with CR, PR, or stable disease (SD)] in the overall population and in subgroups of interest; and description of sociodemographic and clinical characteristics of patients in the overall population and according to subgroups of interest, and treatment characteristics. Response rates are calculated based on clinical assessment or Response Evaluation Criteria in Solid Tumors (RECIST) v1.1 criteria by the study investigator. An independent central review was not conducted for this study. Subgroups of interest include patients stratified by International Metastatic RCC Database Consortium (IMDC) risk group, metastatic sites, baseline comedications, concomitant corticosteroid therapy, concomitant radiation therapy (RT), prior nephrectomy, and nivolumab line of treatment (LOT).

### Safety and tolerability

Another secondary study objective is to describe the incidence and severity of treatment-related adverse events (TRAEs) and time to onset of TRAEs. TRAEs are graded according to the National Cancer Institute Common Terminology Criteria for Adverse Events version 4.8 and are defined as any untoward medical occurrence in a study participant that arises or worsens after the start of nivolumab. Serious TRAEs are defined as any untoward medical occurrence that at any dose is life-threatening, requires inpatient hospitalization/causes prolongation of existing hospitalization, results in persistent or significant disability/incapacity, or results in death. Severity and relation to nivolumab treatment were determined by the treating physician.

### Statistical analysis

As this is a non-comparative observational study, the sample size was estimated based on the desired 95% confidence interval (CI) width. Assuming a 1-year survival rate of 70% (based on CheckMate 025 results),^7^ we determined that a sample size of 323 patients for the nivolumab monotherapy cohort would provide an estimate of the proportion of patients still alive at 1 year with a precision of ±5.0%.

The safety analysis set included patients who had received at least one nivolumab infusion and the effectiveness analysis set included a subpopulation of the safety analysis set who met the inclusion criteria. Patient and clinical characteristics were summarized using descriptive statistics. OS and PFS with their 95% CIs were estimated using the Kaplan–Meier method. Patients were censored at the last record or assessment for those lost to follow-up, those who enrolled into a clinical trial, or died. ORR, DCR, and BOR are reported as rates. Subgroup analyses were conducted in a complete case manner using descriptive statistics. Statistical analyses were carried out using SAS software v9.4 (SAS Institute, Cary, NC).

## Results

### Patients

For this interim analysis, patients were enrolled from January 2018 to December 2019 and were followed up until the database lock date of 18 February 2021. A total of 330 patients were enrolled in the nivolumab cohort, of whom 328 patients were included in the safety analysis set and 325 in the effectiveness analysis set; five patients were excluded (two died before nivolumab initiation and three met the exclusion criteria; Supplementary Figure S1, available at https://doi.org/10.1016/j.esmoop.2024.103602).

The baseline patient characteristics and their prior and concomitant treatments are summarized in Table 1 and are compared with those of the CheckMate 025 study population. The median (range) age was 71 (37-94) years; 30.5% of patients were aged <65 years, 37.5% were aged ≥65 to <75 years, and 32.0% were aged ≥75 years. Of the 325 assessable patients, 38.2% had a Karnofsky score <80 and 69.5% had undergone previous nephrectomy. At nivolumab initiation, 95.1% of patients had clear-cell histology, and a sarcomatoid component was noted in 4.0% of patients. The most common metastatic sites were lung (67.4%), bone (35.7%), and liver (20.6%). Notably, 7.4% of patients had brain metastases and 14.2% of patients had serous and glandular metastases.

**Table 1:** Baseline characteristics and treatment patterns of patients treated with nivolumab after at least one prior systemic therapy in WITNESS and CheckMate 025^7^

Parameter	WITNESS nivolumab cohort (*n* = 325)^a^	CheckMate 025 (*n* = 410)
Age, years		
Median (range)	71 (37-94)	62 (23-88)
Category, *n* (%)		
<65	99 (30.5)	257 (63.0)
≥65 to <75	122 (37.5)	119 (29.0)
≥75	104 (32.0)	34 (8.3)
Sex,^b^*n* (%)		
Male	235 (72.3)	315 (77.0)
Karnofsky performance score, *n* (%)		
<70	59 (18.2)	2 (0.5)
70	65 (20.0)	22 (5.0)
80	90 (27.7)	110 (27.0)
90	56 (17.2)	150 (37.0)
100	45 (13.9)	126 (31.0)
Missing	10 (3.1)	—
IMDC risk group,^c^*n* (%)		
Favorable	19 (5.8)	55 (13.0)
Intermediate	108 (33.2)	242 (59.0)
Poor	79 (24.3)	96 (23.0)
Not assessed	119 (36.6)	—
Histologic subtype at initial diagnosis,^d^*n* (%)		
Clear cell	309 (95.1)	410 (100.0)
Non-clear cell	16 (4.9)	—
Disease duration at baseline, median (range), months	32.7 (2-422.5)	NA
Location of metastases,^e^*n* (%)		
Lung	219 (67.4)	278 (68.0)
Bone	116 (35.7)	76 (19.0)
Liver	67 (20.6)	100 (24.0)
Brain	24 (7.4)	NA^f^
Serous	46 (14.2)	NR
Glandular	46 (14.2)	NR
Sarcomatoid component, *n* (%)		
Yes	13 (4.0)	NA
Missing	39 (12)	NR
Previous nephrectomy, *n* (%)		
Yes	226 (69.5)	364 (89.0)
Number of prior systemic regimens, *n* (%)		
1	253 (77.8)	294 (72.0)
2	72 (22.1)	116 (28.0)
Treatment received before nivolumab, *n* (%)		
Adjuvant therapy		
TKI	20 (6.2)	—
Pazopanib	8 (2.5)	—
Sunitinib	12 (3.7)	—
Anti-VEGF	1 (0.3)	—
First-line therapy		
TKI	288 (88.6)	
Axitinib	2 (0.6)	51 (12.0)^g^
Cabozantinib	2 (0.6)	—
Pazopanib	110 (33.8)	119 (29.0)^g^
Sorafenib	2 (0.6)	—
Sunitinib	175 (53.8)	246 (60.0)^g^
mTOR	6 (1.8)	—
Cytokine	1 (0.3)	—
Anti-VEGF	4 (1.2)	—
Other	3 (0.9)	—
Second-line therapy	*N* = 72	
TKI	62 (86.1)	—
Axitinib	11 (15.3)	—
Cabozantinib	25 (34.7)	—
Pazopanib	11 (15.3)	—
Sorafenib	0	—
Sunitinib	16 (22.2)	—
mTOR	8 (11.1)	—
Cytokine	0	—
Anti-VEGF	0	—
Other	0	—

IMDC, International Metastatic RCC Database Consortium; mTOR, mammalian target of rapamycin; NA, not available; NR, not reported; TKI, tyrosine kinase inhibitors; VEGF, vascular endothelial growth factor.

a Percentages are based upon observed values with missing data excluded from calculations.

b As determined by a set of biological attributes that are associated with physical and physiological features.

c IMDC risk group was derived using prognostic criteria available in electronic case report forms.

d Twelve patients with papillary renal cell carcinoma, 50% of patients had papillary type I.

e Multiple locations per patient possible.

f Patients with central nervous system metastases were excluded from CheckMate 025.

g Received at any prior line of therapy.

At the first nivolumab dose, the median (range) time since initial RCC diagnosis was 32.7 (2-422.5) months. Over three-quarters of the patients (77.8%) received nivolumab as second-line therapy; tyrosine kinase inhibitors were predominantly used before nivolumab therapy.

### Effectiveness

At data cut-off, 256 patients (78.8%) had discontinued nivolumab treatment; the main reasons for discontinuation were disease progression (62.5%), adverse events (AEs; 16.4%, of which 45.2% were considered nivolumab-related), and death (10.2%). Median nivolumab DOT was 3.8 (95% CI 2.9-4.6) months, and median DOR was 16.5 (95% CI 10.9-23.3) months.

**Figure 1:**
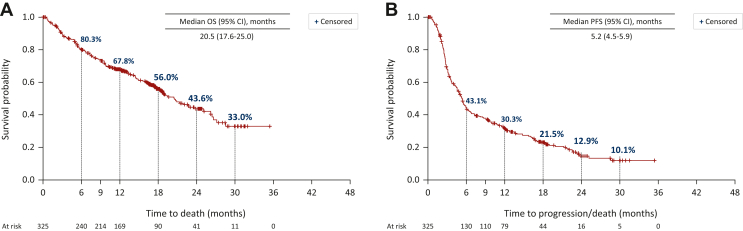
**Overall response and Progression free survival with nivolumab.** Kaplan–Meier plots of (A) OS and (B) PFS in patients treated with nivolumab. CI, confidence interval; OS, overall survival; PFS, progression-free survival.

The median OS was 20.5 (95% CI 17.6-25.0) months, with an estimated survival probability of 67.8% (95% CI 62.5% to 73.1%) at 12 months and 43.6% (95% CI 36.4% to 50.8%) at 24 months (Figure 1A). The median PFS was 5.2 (95% CI 4.5-5.9) months (Figure 1B).

The BOR was CR in 12 patients (3.7%), PR in 80 (24.6%), SD in 109 (33.5%), and progressive disease in 50 (15.4%; Table 2). The ORR was 28.3% and the DCR was 61.8%.

**Table 2:** Best overall response with nivolumab

Best response as per RECIST or clinical assessment, *n* (%)	*N* = 325
CR	12 (3.7)
PR	80 (24.6)
SD	109 (33.5)
PD	50 (15.4)
Missing response/no tumor assessment	45 (13.8)
ORR, *n* (%)	92 (28.3)
DCR, *n* (%)	201 (61.8)

CR, complete response; DCR, disease control rate; ORR, objective response rate; PD, progressive disease; PR, partial response; RECIST, Response Evaluation Criteria in Solid Tumors; SD, stable disease.

#### Effectiveness according to IMDC risk group

At nivolumab initiation, 19 patients (5.8%) had favorable IMDC risk scores, 108 (33.2%) had intermediate risk scores, and 79 (24.3%) had poor risk scores; 119 patients (36.6%) had no IMDC risk score because this is not routinely assessed at second- or third-line treatment (Table 1). When nivolumab effectiveness was evaluated in patients stratified by IMDC risk group, median OS was 26.2 months in those with favorable risk scores, 25.0 months in those with intermediate risk scores, 8.6 months in those with poor risk score, and 20.9 months in those with no IMDC risk score (Supplementary Figure S2, available at https://doi.org/10.1016/j.esmoop.2024.103602). These data suggest that patients with no IMDC risk assessment mostly likely had intermediate risk scores. Median PFS estimates were 5.8, 5.2, and 3.5 months in patients with favorable, intermediate, and poor IMDC risk scores, respectively, and 5.9 months in those with no IMDC risk score.

#### Effectiveness according to metastatic sites

Median OS and PFS were 18.3 and 4.0 months for patients with bone metastases, 13.1 and 3.5 months for brain metastases, and 9.7 and 4.4 months for serous metastases (Supplementary Table S1, available at https://doi.org/10.1016/j.esmoop.2024.103602). The median OS and PFS were longest in patients with glandular metastases (20.5 and 5.1 months, respectively; Supplementary Table S1, available at https://doi.org/10.1016/j.esmoop.2024.103602).

#### Effectiveness according to baseline comedications

All patients received comedications, with 74.5% receiving <5 comedications, 19.1% receiving 5-9 comedications, and 6.5% receiving >9 comedications. Sample sizes were small in the two groups with ≥5 comedications. Baseline characteristics were comparable among the patient groups (Supplementary Table S2, available at https://doi.org/10.1016/j.esmoop.2024.103602), although a higher proportion of patients receiving 5-9 and >9 comedications had a Karnofsky score <80.

The median OS was maintained across groups receiving <5 comedications (21.9 months) and 5-9 comedications (25.0 months), but those receiving >9 comedications had a reduced OS of 13.5 months (Supplementary Table S2, available at https://doi.org/10.1016/j.esmoop.2024.103602). The median PFS was 5.3 months in patients receiving <5 and 5-9 comedications but was reduced in those receiving >9 comedications (3.0 months).

#### Effectiveness according to baseline concomitant corticosteroid therapy

Thirteen patients were identified as receiving concomitant systemic corticosteroids before nivolumab initiation and were analyzed for OS. According to the baseline characteristics of these patients, more than three-quarters of patients in this subgroup (10/13; 76.9%) had a baseline Karnofsky score of ≤70 (Supplementary Table S3, available at https://doi.org/10.1016/j.esmoop.2024.103602). Prednisone was used by five patients (38.5%) and prednisolone and methylprednisolone by four patients (30.8%) each. Corticosteroids were used for the management of the tumor in six patients (46.2%; including brain metastases in three patients), a pre-existing disease in five patients (38.5%; including autoimmune disease in two patients and unknown disease in three patients), or as prophylaxis in two patients (15.4%). Most patients (11/13; 84.6%) were receiving dosages of ≥10 mg/day (prednisone dose equivalent).

The median OS in patients receiving concomitant corticosteroid therapy was 2.6 months, with a survival probability of 33.3% at 12 months (Supplementary Table S3, available at https://doi.org/10.1016/j.esmoop.2024.103602).

#### Effectiveness according to line of treatment

Among the 325 patients, 253 (77.8%) received nivolumab as second-line therapy and 72 (22.2%) as third-line therapy. Baseline characteristics of patients according to nivolumab LOT are given in Supplementary Table S4, available at https://doi.org/10.1016/j.esmoop.2024.103602.

The ORR was 28.9% in patients who received nivolumab as second LOT and 26.4% for third LOT, and the DCR was 64.2% and 56.9%, respectively (Supplementary Table S4, available at https://doi.org/10.1016/j.esmoop.2024.103602). Survival outcomes were similar irrespective of the LOT. Median OS and PFS were 20.9 and 5.3 months, respectively, when nivolumab was given as second LOT and 18.9 and 4.5 months, respectively, for third LOT (Supplementary Table S4, available at https://doi.org/10.1016/j.esmoop.2024.103602).

#### Effectiveness according to nephrectomy status

As mentioned above, 226 of 325 patients (69.5%) had undergone prior nephrectomy. A higher proportion of patients without prior nephrectomy had metastases at diagnosis (88.9% versus 42.0%) and Karnofsky score <80 (47.5% versus 34.1%) compared with patients with prior nephrectomy (Supplementary Table S5, available at https://doi.org/10.1016/j.esmoop.2024.103602). Although most of the IMDC risk data were missing (as it is not mandatory to evaluate IMDC risk scores in second or later LOT), the proportion of patients with intermediate or poor risk scores was approximately twofold higher among patients without prior nephrectomy than in those with prior nephrectomy (50.5% versus 25.2%).

Patients with prior nephrectomy had a higher ORR (33.6% versus 16.2%) and DCR (67.7% versus 48.5%), and longer OS (22.6 versus 16.1 months) and PFS (5.5 versus 4.4 months), compared with those without prior nephrectomy (Supplementary Table S5, available at https://doi.org/10.1016/j.esmoop.2024.103602).

### Safety

**Figure 2:**
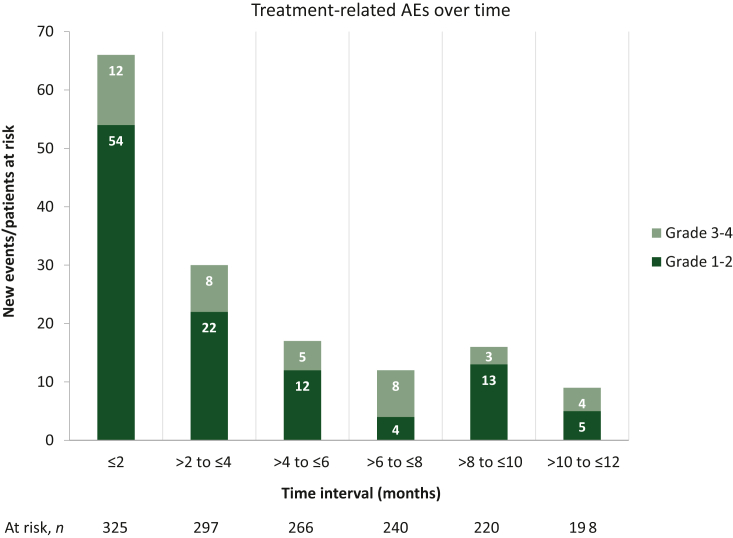
**Change in the proportion of patients with grade 1-2 and grade 3-4 treatment-related AEs every 2 months over 12 months.** AEs, adverse events.

TRAEs of any grade were reported in 32.0% of patients, grade ≥3 TRAEs and serious TRAEs in 11.1% each, and TRAEs leading to discontinuation in 8.9% (Table 3). The median (range) time to onset was 1.5 (0-28.1) months for first TRAE and 4.0 (0.2-15.9) months for grade ≥3 TRAEs. The majority of TRAEs of any grade were reported within 2 months of initiating nivolumab treatment, with the number of TRAEs decreasing gradually over time (Figure 2). The most common TRAEs were asthenia (*n* = 29; 11.5%), pruritus (*n* = 22; 8.7%), and diarrhea (*n* = 14; 5.6%), and the most common grade ≥3 TRAE was asthenia (*n* = 10; 18.5%; Supplementary Table S6, available at https://doi.org/10.1016/j.esmoop.2024.103602). TRAEs leading to discontinuation were hepatocellular injury and blood alkaline phosphate increased (each *n* = 3; 7.7%) followed by anemia, nausea, malignant neoplasm progression, and general physical health deterioration (each *n* = 2; 5.1%). Serious TRAEs included malignant neoplasm progression (*n* = 4; 9.1%) and diarrhea (*n* = 3; 6.8%). Two deaths occurred due to serious TRAEs; both patients died from malignant neoplasm progression.

**Table 3:** Treatment-related adverse events in the nivolumab cohort

TRAEs	Nivolumab cohort (*N* = 325)
Any grade, *n* (%)	104 (32.0)
Grade 1	64 (19.7)
Grade 2	40 (12.3)
Grade 3-4	36 (11.1)
Grade 3-4 in first 12 months	34 (10.5)
Leading to discontinuation of nivolumab	29 (8.9)
Serious TRAEs	36 (11.1)
Time to onset of first TRAE (all grades), median (range), months	1.5 (0-28.1)
Time to onset of first TRAE (grade 3 or 4), median (range), months	4.0 (0.2-15.9)

TRAEs, treatment-related adverse events.

#### AEs in patients receiving concomitant radiation therapy

In total, 31 patients received RT targeting metastases during nivolumab treatment, and 35 metastatic sites were irradiated. Irradiated sites were mainly located in the bone (64.5%), brain (16.1%), and lungs (12.9%; Supplementary Table S7, available at https://doi.org/10.1016/j.esmoop.2024.103602).

The safety profile of nivolumab in patients who received concomitant RT is summarized in Supplementary Table S7, available at https://doi.org/10.1016/j.esmoop.2024.103602. Seven patients (21.9%) developed at least one RT-related AE within 3 months after the first cycle of RT during nivolumab treatment, including three cases of pruritus.

## Discussion

This interim analysis of cohort 1 in the WITNESS study showed that nivolumab monotherapy is effective and generally well tolerated in previously treated patients with aRCC in the real-world setting. These findings are consistent with those of the pivotal phase III CheckMate 025 study,^7^ and are promising considering that patients in our study were more frail and had a poorer performance status than those enrolled in CheckMate 025. Patients included in our study were of higher median age (71 versus 62 years), poorer performance status (38.2% versus 5.5% in patients with Karnofsky score <80), and a higher proportion of patients had bone metastases (35.7% versus 18.5%) compared with CheckMate 025.^7^ Our study also included patients with non-clear-cell aRCC, and brain metastases, a population that was excluded from CheckMate 025 (see comparison in Table 1).

The median OS and PFS of the overall population were 20.5 and 5.2 months, respectively, and were comparable with those of CheckMate 25 (25.0 and 4.6 months, respectively).^7^ The 1- and 2-year OS rates were 67.8% and 43.6% in our study versus 76.0% and 52.0% in CheckMate 025, respectively.^10^ However, more patients achieved a CR (3.7%) in our study, resulting in an ORR of 28.3% compared with CheckMate 025 (CR: 1.0%; ORR: 26.0%).

Several other studies conducted in Germany,^11^ France,^12^ and Italy^13^ have investigated the benefit of nivolumab treatment in patients with aRCC in a real-world setting. Our results support the findings from these studies, which have shown the effectiveness of nivolumab in a broad range of patients, including older and heavily pre-treated patients and those with advanced disease. Although a higher proportion of patients had undergone prior nephrectomy in the German real-world NORA study of 228 patients with aRCC^11^ (86.9% versus 69.5% in our study), the OS and PFS were consistent with our study [24.3 (95% CI 19-28) months and 5.3 (95% CI 3.9-6.7) months, respectively, in NORA versus 20.5 (95% CI 17.6-25.0) months and 5.2 (95% CI 4.5-5.9) months, respectively, in our study]. Tumor response to nivolumab treatment was better in our study than in the NORA study; only 1.3% of patients in NORA achieved a CR, and the ORR was 20.0%.^11^ The NIVOREN GETUG AFU 26 study in 720 patients with clear-cell aRCC was one of the largest French multicenter prospective studies of nivolumab in the real-world setting.^12^ This study included 83 patients (12.3%) with brain metastases [compared with 24 patients (7.4%) in our study]. Unlike our study, patients with non-clear-cell aRCC were excluded from NIVOREN. After a follow-up of 20.9 months, the median PFS was 3.2 months, the 1-year OS rate was 69.0%, and ORR was 20.8%. Notably, 46.1% of patients continued treatment beyond disease progression.^12^ The Italian Nivolumab Renal Cell Cancer Expanded Access Program (IEAP) was a real-world cohort study in 389 patients with metastatic RCC who received nivolumab treatment.^13^ Patient clinical characteristics in the IEAP study were broadly similar to those in our nivolumab cohort. Although median OS was not reached in the IEAP study, the 1-year survival rate (63.0%) was comparable with our study (67.8%). This was despite a higher proportion of patients with favorable IMDC risk scores in the IEAP study (20.2% versus 5.8% in our study).

In the CheckMate 025 study, the trend for OS and ORR benefit with nivolumab compared with everolimus in the overall population was sustained across multiple subgroups, including those defined by prognostic risk, age, number and sites of metastases, and prior therapies, without specific safety concerns.^14^ Based on these results, clinical practice guidelines have recommended nivolumab as the new standard of care for a broad range of patients with previously treated aRCC.^15^, ^16^, ^17^ Similarly, we found that nivolumab monotherapy was effective in patients with bone or glandular metastases, patients receiving multiple comedications and corticosteroids at baseline, and irrespective of the LOT.

A subgroup analysis focusing on the location of metastasis was carried out in our study for three reasons: firstly, response to nivolumab may vary depending on the site of metastasis; secondly, there is a lack of real-world evidence to objectively confirm physicians’ choice of second- and third-line treatment (immunotherapy or tyrosine kinase inhibitors) in patients with metastases; and lastly, there is limited information about treatment efficacy for brain, bone, serous, and glandular metastases, because either these patients are usually excluded from large, randomized phase III trials or specific subgroup analyses are not conducted due to limited sample size. A retrospective study by Negishi and colleagues was the first study to evaluate tumor response to nivolumab in different metastatic and primary sites in patients with aRCC, and reported that, compared with the overall patient population, response rates were worse in patients with bone and brain metastases and OS was shorter in patients with lung or liver metastases.^18^ In our study, the presence of brain or serous metastases negatively impacted OS, while OS for bone or glandular metastases was broadly similar to that of the overall population. This site-specific response to nivolumab could be attributed to the tumor microenvironment, which differs between the primary organ and sites of metastasis, and also between different sites of metastasis.^19^

Compared with the overall population, median OS and PFS were shorter in patients on concomitant corticosteroids or more than nine comedications when nivolumab was started; however, these data should be viewed with caution due to the small size of this patient subgroup and the high proportion of patients with poor performance status (i.e. Karnofsky score <80).

The safety results of our study were consistent with the known safety profile of nivolumab; no new safety signals were identified. The proportion of patients with any-grade TRAEs and that of patients with grade 3-4 TRAEs with nivolumab were much lower in the current study (32% and 11.1%, respectively) than in the nivolumab arm in CheckMate 025 (79.0% and 19.0%, respectively).^7^ Overall, our data support the manageable toxicity of nivolumab seen in other real-world studies.^11^, ^12^, ^13^ Moreover, concomitant RT to metastatic sites during nivolumab treatment appeared to be a feasible treatment strategy in this real-world setting, with no new safety signals observed.

The combination of nivolumab plus ipilimumab is currently being evaluated in a separate cohort of the WITNESS study. This combination was approved for the treatment of adult patients with intermediate-/poor-risk aRCC, based on the clinical efficacy and manageable safety profile of nivolumab plus ipilimumab demonstrated in the phase III CheckMate 214 trial.^20^

The strength of our study lies in the inclusion of patients with a broad range of demographic and pretreatment features, attributable to our minimal selection criteria, thus providing for a high level of external validity. However, our study has some limitations, mainly related to the nature of non-comparative, observational research. These include risk of selection, information, and attrition biases, missing data, measurement error and misclassification, and risk of under- or over-recruitment. In particular, the lack of independent central review in this study limited our ability to standardize pathology, imaging, and response rates as per RECIST or clinical assessment across study centers. However, this methodological approach reflects the current state of real-world clinical practice, and only highlights the demonstrable effectiveness and safety of nivolumab among patients with aRCC treated in France.

### Conclusions

The interim results of the WITNESS study show that nivolumab monotherapy is an effective and tolerable treatment option for patients with aRCC, irrespective of their disease characteristics and LOT.

## Figures and Tables

**Supplementary Figure:**