# Supplementary material for: Nivolumab in patients with advanced renal cell carcinoma in France: interim results of the observational, real-world WITNESS study
Source: ESMO Open. 2024 Jun 18;9(7):103602. doi: 10.1016/j.esmoop.2024.103602 (PMC11237687; doi:10.1016/j.esmoop.2024.103602)
Supplement: Supplementary data [file mmc1.docx]

**Nivolumab in patients with advanced renal cell carcinoma in France: interim results of the observational, real-world WITNESS study**

**Supplementary Material**

**Authors:** Philippe Barthélémy^a^, Laurence Albigès^b^, Bernard Escudier^b^, Bérengère Narciso^c^, Pierre Bigot^d^, Mohamad Chehimi^e^, Sheik Emambux^f^, Fabien Calcagno^g^, Guillaume Mouillet^g^, Jean-Christophe Eymard^h^, Friederike Schlürmann^i^, Sébastien Bailly^j^, Delphine Garbay^k^, Jean-François Berdah^l^, Marjorie Baciuchka Palmaro^m^, Marine Gross Goupil^n^, Dominique Spaeth^o^, Sonia Néré^p^, Carole Quentric^p^ Yann-Alexandre Vano^q^, Antoine Thiery-Vuillemin^g^

**Affiliations:** ^a^Institut de Cancérologie Strasbourg Europe, Strasbourg, France; ^b^Gustave Roussy Cancer Campus, Villejuif, Paris, France; ^c^Centre Hospitalier Universitaire de Tours, Tours CEDEX 9, France; ^d^Centre Hospitalier Universitaire d’Angers, Angers CEDEX 9, France; ^e^Centre Hospitalier de Saint-Quentin, Saint-Quentin, France; ^f^Centre Hospitalier Universitaire de Poitiers, Poitiers, France; ^g^Centre Hospitalier Universitaire de Besançon, Besançon, France; ^h^Institut Jean Godinot, Reims CEDEX, France; ^i^Centre Hospitalier Intercommunal Quimper, Quimper, France; ^j^Centre Hospitalier Universitaire de Clermont-Ferrand, France; ^k^Clinique Tivoli-Ducos, Bordeaux, France; ^l^Hôpital privé Toulon Hyères - Sainte Marguerite, Hyères, France; ^m^Hôpital Nord, Marseille, France; ^n^Centre Hospitalier Universitaire de Bordeaux, Hôpital Saint-André, Bordeaux CEDEX, France; ^o^Polyclinique de Gentilly, Nancy, France; ^p^Bristol Myers Squibb, Rueil-Malmaison, Paris, France; ^q^Hôpital Européen Georges Pompidou, APHP Centre – Université de Paris, Paris, France.

**Corresponding author**: Philippe Barthélémy

Email: [p.barthelemy@icans.eu](mailto:p.barthelemy@icans.eu)

**Supplementary Figure S1.**

**Patient disposition.**


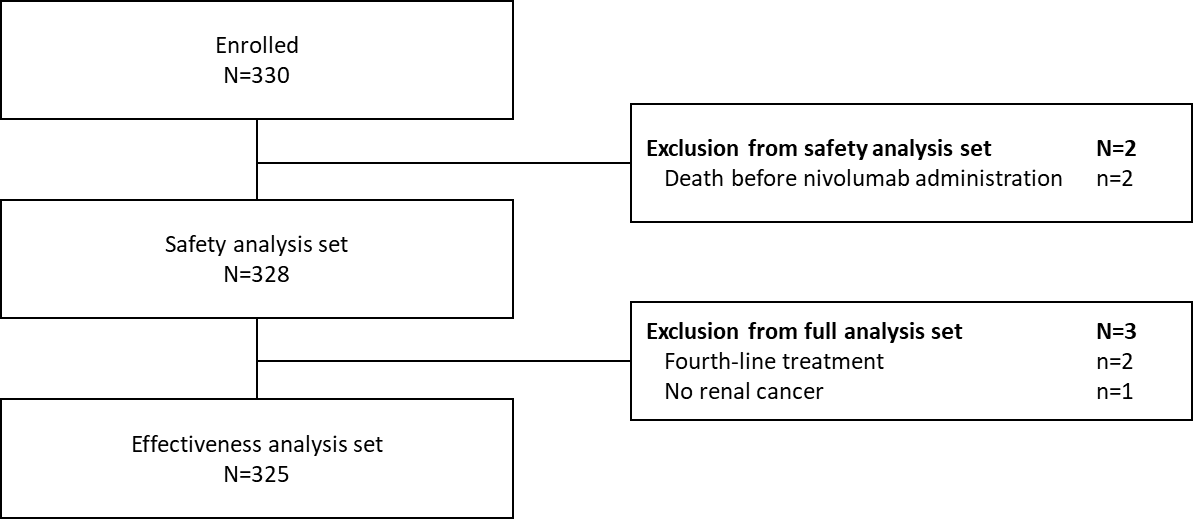


**Supplementary Figure S2.**

**Kaplan-Meier plot of overall survival in patients stratified by International Metastatic RCC Database Consortium (IMDC) risk group at nivolumab initiation.**


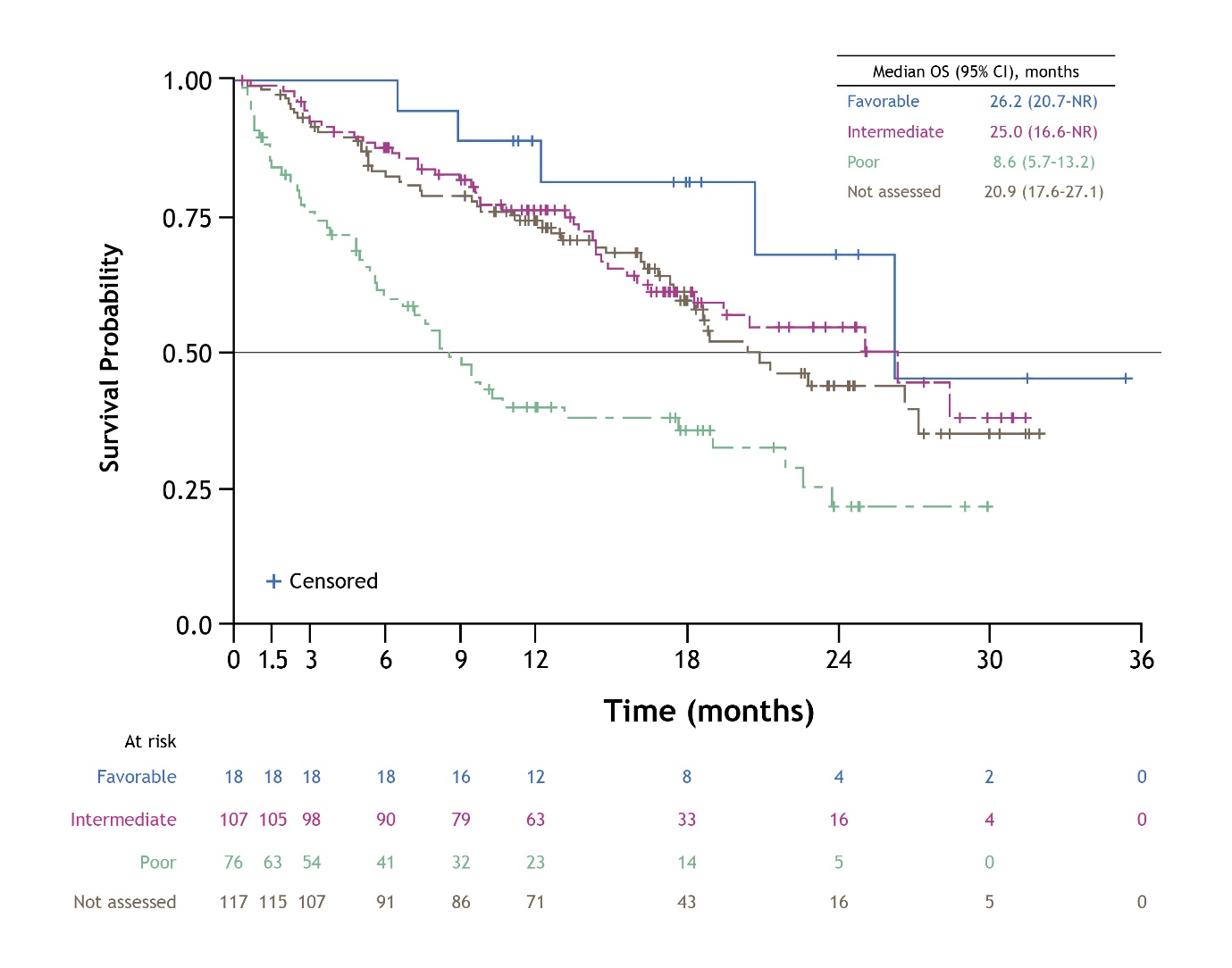
CI, confidence interval; NR, not reached; OS, overall survival.

**Supplementary Table S1.**

**Effectiveness outcomes according to metastasis sites**

|  | **Metastasis site** | | | |
| --- | --- | --- | --- | --- |
|  | **Brain**  **(n=24)** | **Bone**  **(n=116)** | **Serous**  **(n=46)** | **Glandular**  **(n=46)** |
| **OS** |  |  |  |  |
| Median OS (95% CI), months | 13.1 (5.5–17.3) | 18.3 (13.2–21.9) | 9.7 (5.7–20.5) | 20.5 (9.7–NR) |
| Estimated OS rate, % (95% CI) |  |  |  |  |
| 6 months | 68.8 (45.0–84.0) | 77.1 (68.0–84.0) | 64.9 (49.0–77.0) | 80.9 (65.0–90.0) |
| 12 months | 58.9 (36.0–76.0) | 64.2 (54.0–73.0) | 44.9 (29.0–59.0) | 56.8 (40.0–71.0) |
| 24 months | 21.2 (6.0–43.0) | 34.0 (22.0–46.0) | 21.0 (6.0–41.0) | 44.0 (26.0–61.0) |
| **PFS** |  |  |  |  |
| Median PFS (95% CI), months | 3.5 (2.5–5.4) | 4.0 (3.1–5.3) | 4.4 (2.4–6.6) | 5.1 (3.1–10.7) |
| Estimated PFS rate, % (95% CI) |  |  |  |  |
| 6 months | 29.0 (12.0–48.0) | 33.8 (25.0–43.0) | 36.3 (22.0–51.0) | 40.6 (26.0–55.0) |
| 12 months | 19.3 (6.0–38.0) | 23.2 (16.0–32.0) | 25.9 (14.0–40.0) | 29.9 (17.0–44.0) |
| 24 months | 6.4 (0–24.0) | 7.1 (3.0–15.0) | 9.9 (1.0–30.0) | 12.8 (4.0–28.0) |

CI, confidence interval; NR, not reached; OS, overall survival; PFS, progression-free survival.

**Supplementary Table S2.**

**Baseline characteristics and effectiveness outcomes according to the number of comedications received at baseline and during the study (N=325)**

| **Baseline characteristics** | **Number of comedications** | | |
| --- | --- | --- | --- |
|  | **<5**  **(n=242)** | **5–9**  **(n=62)** | **>9**  **(n=21)** |
| Age |  |  |  |
| Median (range), years | 69 (37–89) | 73 (50–94) | 74 (51–82) |
| ≥75 years, n (%) | 68 (28.1) | 27 (43.5) | 9 (42.9) |
| Sex^a^, n (%) |  |  |  |
| Male | 179 (74.0) | 41 (66.1) | 15 (71.4) |
| Karnofsky score, n (%) |  |  |  |
| ≤70 | 79 (32.6) | 33 (53.2) | 12 (57.1) |
| 80–100 | 157 (64.9) | 28 (45.2) | 6 (28.6) |
| Missing | 6 (2.5) | 1 (1.6) | 3 (14.2) |
| Metastatic site, n (%) |  |  |  |
| Lung | 166 (69.5) | 41 (67.2) | 12 (57.1) |
| Bone | 86 (36.0) | 19 (31.1) | 11 (52.4) |
| Liver | 49 (20.5) | 13 (21.3) | 5 (23.8) |
| Brain | 17 (7.1) | 4 (6.6) | 3 (14.3) |
| Number of prior therapies, n (%) |  |  |  |
| 1 | 191 (78.9) | 47 (75.8) | 15 (71.4) |
| 2 | 51 (21.1) | 15 (24.2) | 6 (28.6) |
| Histology, n (%) |  |  |  |
| Clear cell RCC | 231 (95.5) | 58 (93.5) | 20 (95.2) |
| Nephrectomy, n (%) | 173 (71.5) | 39 (62.9) | 14 (66.7) |
| **Effectiveness** | | | |
| Median OS (95% CI), months | 21.9 (18.9–NR) | 25.0 (10.7–28.4) | 13.5 (7.5–18.3) |
| Estimated OS rate, % (95% CI) |  |  |  |
| 6 months | 82.1 (77.1–87.1) | 74.9 (63.9–85.9) | 76.2 (58.0–94.4) |
| 12 months | 70.2 (64.1–76.2) | 61.2 (48.7–73.6) | 61.0 (39.7–82.2) |
| 18 months | 59.6 (52.6–66.7) | 51.5 (37.8–65.2) | 33.2 (11.9–54.6) |
| 24 months | 45.2 (36.5–54.0) | 51.5 (37.8–65.2) | 0 |
| 30 months | 40.3 (30.2–50.4) | 22.5 (2.6–42.4) | 0 |
| Median PFS (95% CI), months | 5.3 (4.5–6.6) | 5.3 (3.6–9.0) | 3.0 (2.3–4.9) |
| Estimated PFS rate, % (95% CI) |  |  |  |
| 6 months | 46.0 (39.4–52.5) | 42.6 (29.9–55.2) | 14.3 (0–29.3) |
| 12 months | 30.8 (24.6–37.0) | 35.6 (23.3–47.8) | 9.5 (0–22.1) |
| 18 months | 23.4 (17.4–29.4) | 20.2 (8.3–32.0) | – |
| 24 months | 12.5 (6.7–18.4) | 16.8 (5.3–28.4) | – |
| 30 months | 11.0 (5.1–16.8) | – | – |

CI, confidence interval; NR, not reached; OS, overall survival; PFS, progression-free survival; RCC, renal cell carcinoma.
^a^As determined by a set of biological attributes that are associated with physical and physiological features.

**Supplementary Table S3.**

**Baseline characteristics and effectiveness outcomes in patients receiving concomitant corticosteroid therapy at baseline (N=13)**

| **Baseline characteristics** | **N=13** |
| --- | --- |
| Age |  |
| Median (range), years | 70 (54–85) |
| ≥75 years, n (%) | 2 (15.4) |
| Sex^a^, n (%) |  |
| Male | 10 (76.9) |
| Female | 3 (23.1) |
| Type of corticosteroid, n (%) |  |
| Methylprednisolone | 4 (30.8) |
| Prednisolone | 4 (30.8) |
| Prednisone | 5 (38.5) |
| Dose of corticosteroid (prednisone dose equivalent), n (%) |  |
| <10 mg/day | 2 (15.4) |
| ≥10 mg/day | 11 (84.6) |
| Indication for corticosteroid, n (%) |  |
| Pre-existing disease | 5 (38.5) |
| Management of tumor | 6 (46.2) |
| Prophylaxis | 2 (15.4) |
| Karnofsky score, n (%) |  |
| ≤70 | 10 (76.9) |
| 80–100 | 2 (15.4) |
| Data missing | 1 (8.3) |
| Metastatic site, n (%) |  |
| Lung | 9 (69.2) |
| Bone | 6 (46.2) |
| Liver | 5 (38.5) |
| Brain | 3 (23.1) |
| Nephrectomy, n (%) | 7 (53.8) |
| **Effectiveness** | |
| Median OS (95% CI), months | 2.6 (0.8–13.7) |
| Estimated OS rate, % (95% CI) |  |
| 6 months | 41.7 (13.8–69.6) |
| 12 months | 33.3 (6.7–60.0) |
| Median PFS (95% CI), months | 2.6 (0.7–5.3) |
| Estimated PFS rate, % (95% CI) |  |
| 6 months | 25.0 (0.5–49.5) |
| 12 months | 16.7 (0–37.8) |

CI, confidence interval; CR, complete response; DCR, disease control rate; ORR, objective response rate; OS, overall survival; PFS, progression-free survival; PR, partial response; SD, stable disease.
^a^As determined by a set of biological attributes that are associated with physical and physiological features.

**Supplementary Table S4.**

**Baseline characteristics and effectiveness outcomes according to nivolumab line of treatment**

| **Baseline characteristics** | **Nivolumab LOT** | |
| --- | --- | --- |
|  | **Second line**  **(n=253)** | **Third line**  **(n=72)** |
| Age at nivolumab initiation, median (range), years | 71 (38–94) | 69 (37–87) |
| Sex^a^, n (%) |  |  |
| Male | 188 (74.3) | 47 (65.3) |
| Female | 65 (25.7) | 25 (34.7) |
| Karnofsky score, n (%) |  |  |
| ≤70 | 89 (35.2) | 35 (48.6) |
| 80–100 | 156 (61.7) | 35 (48.6) |
| Missing | 8 (3.2) | 2 (2.8) |
| Number of metastatic sites |  |  |
| 0 | 2 (0.8) | 2 (2.8) |
| 1 | 62 (24.5) | 12 (16.7) |
| 2 | 73 (28.9) | 22 (30.6) |
| 3–6 | 103 (40.7) | 30 (41.7) |
| >6 | 13 (5.1) | 6 (8.3) |
| **Effectiveness** | | |
| Best response per RECIST, n (%) |  |  |
| CR | 11 (4.3) | 1 (1.4) |
| PR | 62 (24.5) | 18 (25.0) |
| SD | 87 (34.4) | 22 (30.6) |
| PD | 37 (14.6) | 13 (18.1) |
| Missing | 1 (0.4) | 1 (1.4) |
| ORR, n (%) | 73 (28.9) | 19 (26.4) |
| DCR, n (%) | 160 (64.2) | 41 (56.9) |
| Median OS (95% CI), months | 20.9 (17.6–27.1) | 18.9 (13.5–23.8) |
| Estimated OS rate, % (95% CI) |  |  |
| 6 months | 82.8 (78.0–87.6) | 72.1 (61.7–82.5) |
| 12 months | 69.1 (63.1–75.1) | 63.4 (52.2–74.6) |
| 24 months | 46.7 (38.6–54.9) | 33.5 (18.7–48.4) |
| Median PFS (95% CI), months | 5.3 (4.5–6.2) | 4.5 (3.0–6.3) |
| Estimated PFS rate, % (95% CI) |  |  |
| 6 months | 44.2 (37.8–50.6) | 39.4 (28.1–50.8) |
| 12 months | 31.6 (25.5–37.7) | 26.0 (15.6–36.4) |
| 24 months | 14.5 (8.7–20.4) | 7.5 (0–15.8) |

CI, confidence interval; CR, complete response; DCR, disease control rate; LOT, line of treatment; ORR, objective response rate; OS, overall survival; PD, progressive disease; PFS, progression-free survival; PR, partial response; RECIST, Response Evaluation Criteria in Solid Tumors; SD, stable disease.
^a^As determined by a set of biological attributes that are associated with physical and physiological features.

**Supplementary Table S5.**

**Baseline characteristics and effectiveness outcomes in patients with or without prior nephrectomy at baseline**

| **Baseline characteristics** | **Nephrectomy** | |
| --- | --- | --- |
|  | **Yes**  **(n=226)** | **No**  **(n=99)** |
| Age |  |  |
| Median (range), years | 70 (37–89) | 72 (39–94) |
| ≥75 years, n (%) | 70 (31.0) | 34 (34.3) |
| Sex^a^, n (%) |  |  |
| Male | 165 (73.0) | 70 (70.7) |
| Karnofsky score, n (%) |  |  |
| ≤70 | 77 (34.1) | 47 (47.5) |
| 80–100 | 141 (62.4) | 50 (50.5) |
| Missing | 8 (3.5) | 2 (2.0) |
| Metastasis at diagnosis, n (%) |  |  |
| Yes | 95 (42.0) | 88 (88.9) |
| Metastatic site, n (%) |  |  |
| Lung | 155 (69.5) | 64 (65.3) |
| Bone | 82 (36.8) | 34 (34.7) |
| Liver | 48 (21.5) | 19 (19.4) |
| Brain | 14 (6.3) | 10 (10.2) |
| IMDC risk group, n (%) |  |  |
| Favorable | 18 (8) | 1 (1) |
| Intermediate | 8437.2) | 24 (24.2)) |
| Poor | 38 (16.8) | 41 (41.4)) |
| Not assessed | 86 (38.1) | 33 (33.3) |
| Number of prior therapies, n (%) |  |  |
| 1 | 171 (75.6) | 82 (82.8) |
| 2 | 55 (24.3) | 17 (17.0) |
| Histology, n (%) |  |  |
| Clear cell RCC | 219 (96.9) | 90 (90.9) |
| **Effectiveness** | | |
| Best response per RECIST, n (%) |  |  |
| CR | 10 (4.4) | 2 (2.0) |
| PR | 66 (29.2) | 14 (14.1) |
| SD | 77 (34.1) | 32 (32.3) |
| PD | 28 (12.4) | 22 (22.2) |
| Not evaluated/missing | 45 (19.9)^b^ | 29 (29.3)^c^ |
| ORR, n (%) | 76 (33.6) | 16 (16.2) |
| DCR, n (%) | 153 (67.7) | 48 (48.5) |
| Median OS (95% CI), months | 22.6 (18.9–28.4) | 16.1 (9.4–20.9) |
| Estimated OS rate, % (95% CI) |  |  |
| 6 months | 84.5 (79.7–89.3) | 70.0 (60.5–79.5) |
| 12 months | 72.8 (66.8–78.8) | 55.6 (45.1–66.1) |
| 18 months | 61.5 (54.4–68.6) | 42.7 (31.4–54.0) |
| 24 months | 49.1 (40.7–57.6) | 30.3 (17.0–43.5) |
| 30 months | 37.2 (25.9–48.4) | 22.7 (6.4–39.0) |
| Median PFS (95% CI), months | 5.5 (4.8–7.2) | 4.4 (2.9–5.4) |
| Estimated PFS rate, % (95% CI) |  |  |
| 6 months | 46.8 (40.1–53.5) | 34.3 (24.4–44.2) |
| 12 months | 33.6 (27.2–40.1) | 22.3 (13.5–31.1) |
| 18 months | 24.8 (18.5–31.0) | 13.1 (4.8–21.3) |
| 24 months | 16.3 (10.0–22.7) | 4.4 (0–10.0) |
| 30 months | 12.2 (5.3–19.2) | - |

CI, confidence interval; CR, complete response; DCR, disease control rate; IMDC, International Metastatic RCC Database Consortium; ORR, objective response rate; OS, overall survival; PFS, progression-free survival; PD, progressive disease; PR, partial response; RCC, renal cell carcinoma; RECIST, Response Evaluation Criteria in Solid Tumors; SD, stable disease.
^a^As determined by a set of biological attributes that are associated with physical and physiological features.
^b^40 patients were not evaluated, 5 patients had missing data.
^c^24 patients were not evaluated, 5 patients had missing data.

**Supplementary Table S6.**

**All treatment-related adverse events (TRAEs), Grade ≥3 TRAEs, serious TRAEs, and TRAEs leading to discontinuation with an incidence of ≥1.0% among patients treated with nivolumab monotherapy for advanced renal cell carcinoma after ≥1 prior treatment line**

| **System organ class**  **Preferred term, n (%)** | **All TRAEs**  **(n=252)** | **Grade ≥3 TRAEs**  **(n=54)** | **TRAEs leading to discontinuation**  **(n=39)** | **Serious TRAEs**  **(n=44)** |
| --- | --- | --- | --- | --- |
| Blood and lymphatic system disorders | 5 (2.0) | 2 (3.7) | 2 (5.1) | 1 (2.3) |
| Anemia | 3 (1.2) | 2 (3.7) | 2 (5.1) | 1 (2.3) |
| Endocrine disorders | 18 (7.1) | 3 (5.6) | 2 (5.1) | 2 (4.6) |
| Hypothyroidism | 10 (4.0) | 1 (1.9) | 0 | 0 |
| Hyperthyroidism | 6 (2.4) | 1 (1.9) | 1 (2.6) | 1 (2.3) |
| Endocrine disorder | 1 (0.4) | 1 (1.9) | 0 | 1 (2.3) |
| Hypercalcemia of malignancy | 1 (0.4) | 0 | 1 (2.6) | 0 |
| Eye disorders | 5 (2.0) | 3 (5.6) | 1 (2.6) | 2 (4.6) |
| Uveitis | 2 (0.8) | 2 (3.7) | 0 | 1 (2.3) |
| Papilloedema | 1 (0.4) | 1 (1.9) | 1 (2.6) | 1 (2.3) |
| Gastrointestinal disorders | 42 (16.7) | 8 (14.8) | 4 (10.3) | 6 (13.6) |
| Diarrhea | 14 (5.6) | 2 (3.7) | 1 (2.6) | 3 (6.8) |
| Nausea | 9 (3.6) | 1 (1.9) | 2 (5.1) | 0 |
| Vomiting | 6 (2.4) | 2 (3.7) | 0 | 1 (2.3) |
| Constipation | 5 (2.0) | 0 | 0 | 0 |
| Abdominal pain | 3 (1.2) | 0 | 0 | 0 |
| Duodenitis | 1 (0.4) | 1 (1.9) | 0 | 1 (2.3) |
| Pancreatitis | 1 (0.4) | 1 (1.9) | 1 (2.6) | 1 (2.3) |
| Pancreatitis acute | 1 (0.4) | 1 (1.9) | 0 | 0 |
| General disorders and administration site conditions | 41 (16.3) | 12 (22.2) | 3 (7.7) | 2 (4.6) |
| Asthenia | 29 (11.5) | 10 (18.5) | 1 (2.6) | 0 |
| Fatigue | 3 (1.2) | 0 | 0 | 0 |
| General physical health deterioration | 1 (0.8) | 2 (3.7) | 2 (5.1) | 2 (4.6) |
| Hepatobiliary disorders | 7 (2.8) | 5 (9.3) | 5 (12.8) | 3 (6.8) |
| Hepatocellular injury | 3 (1.2) | 2 (3.7) | 3 (7.7) | 0 |
| Hepatic failure | 1 (0.4) | 1 (1.9) | 1 (2.6) | 1 (2.3) |
| Hepatitis | 1 (0.4) | 1 (1.9) | 1 (2.6) | 1 (2.3) |
| Hepatotoxicity | 1 (0.4) | 1 (1.9) | 0 | 1 (2.3) |
| Investigations | 11 (4.4) | 2 (3.7) | 5 (12.8) | 0 |
| Blood alkaline phosphate increased | 5 (2.0) | 1 (1.9) | 3 (7.7) | 0 |
| Aspartate aminotransferase increased | 2 (0.8) | 0 | 1 (2.6) | 0 |
| Gamma-glutamyltransferase increased | 1 (0.4) | 1 (1.9) | 0 | 0 |
| General physical condition | 0 | 0 | 1 (2.6) | 0 |
| Metabolism and nutrition disorders | 9 (3.6) | 3 (5.6) | 4 (10.3) | 3 (6.8) |
| Decreased appetite | 2 (0.8) | 1 (1.9) | 1 (2.6) | 1 (2.3) |
| Cell death | 1 (0.4) | 0 | 1 (2.6) | 0 |
| Hyperglycemia | 1 (0.4) | 1 (1.9) | 1 (2.6) | 1 (2.3) |
| Diabetic metabolic decompensation | 1 (0.4) | 1 (1.9) | 1 (2.6) | 1 (2.3) |
| Musculoskeletal and connective tissue disorders | 20 (7.9) | 3 (5.6) | 3 (7.7) | 6 (13.6) |
| Arthralgia | 10 (4.0) | 1 (1.9) | 1 (2.6) | 2 (4.6) |
| Myalgia | 3 (1.2) | 0 | 0 | 0 |
| Joint pain | 2 (0.8) | 1 (1.9) | 1 (2.6) | 2 (4.6) |
| Polyarthralgia | 1 (0.4) | 1 (1.9) | 1 (2.6) | 1 (2.3) |
| Musculoskeletal pain | 1 (0.4) | 0 | 0 | 1 (2.3) |
| Neoplasms benign, malignant and unspecified (including cysts and polyps) | 7 (2.8) | 1 (1.9) | 2 (5.1) | 4 (9.1) |
| Malignant neoplasm progression | 6 (2.4) | 1 (1.9) | 2 (5.1) | 4 (9.1) |
| Nervous system disorders | 11 (4.4) | 0 | 0 | 0 |
| Paresthesia | 3 (1.2) | 0 | 0 | 0 |
| Renal and urinary disorders | 8 (3.2) | 6 (11.1) | 1 (2.6) | 7 (15.9) |
| Acute kidney injury | 2 (0.8) | 2 (3.7) | 0 | 2 (4.6) |
| Hyponatremia | 2 (0.8) | 1 (1.9) | 1 (2.6) | 1 (2.3) |
| Autoimmune nephritis | 1 (0.4) | 1 (1.9) | 0 | 1 (2.3) |
| Nephritis | 1 (0.4) | 1 (1.9) | 0 | 1 (2.3) |
| Renal failure | 1 (0.4) | 0 | 0 | 1 (2.3) |
| Renal tubular necrosis | 1 (0.4) | 1 (1.9) | 0 | 1 (2.3) |
| Respiratory, thoracic and mediastinal disorders | 10 (4.0) | 0 | 1 (2.6) | 1 (2.3) |
| Cough | 4 (1.6) | 0 | 0 | 0 |
| Lung disorder | 1 (0.4) | 0 | 1 (2.6) | 1 (2.3) |
| Skin and subcutaneous tissue disorders | 45 (17.9) | 2 (3.7) | 2 (5.1) | 1 (2.3) |
| Pruritus | 22 (8.7) | 1 (1.9) | 1 (2.6) | 0 |
| Mucocutaneous rash | 4 (1.6) | 0 | 0 | 0 |
| Dry skin | 3 (1.2) | 0 | 0 | 0 |
| Erythema | 3 (1.2) | 0 | 1 (2.6) | 0 |
| Rash | 3 (1.2) | 0 | 0 | 0 |
| Rash maculo-papular | 2 (0.8) | 1 (1.9) | 0 | 1 (2.3) |
| Psychiatric disorders | 1 (0.4) | 1 (1.9) | 1 (2.6) | 1 (2.3) |
| Confusional state | 1 (0.4) | 1 (1.9) | 1 (2.6) | 1 (2.3) |
| Injury, poisoning and procedural complications | 3 (1.2) | 1 (1.9) | 1 (2.6) | 2 (4.6) |
| Post-radiation pneumonitis | 1 (0.4) | 0 | 1 (2.6) | 1 (2.3) |
| Infusion related reaction | 2 (0.8) | 1 (1.9) | 0 | 1 (2.3) |
| Infections and infestations | 4 (1.6) | 1 (1.9) | 0 | 3 (6.8) |
| Conjunctivitis | 1 (0.4) | 0 | 0 | 1 (2.3) |
| Pneumonia | 1 (0.4) | 0 | 0 | 1 (2.3) |
| Pulmonary sepsis | 1 (0.4) | 1 (1.9) | 0 | 1 (2.3) |
| Cardiac disorders | 2 (0.8) | 1 (1.9) | 2 (5.1) | 0 |
| Cardiomyopathy | 1 (0.4) | 1 (1.9) | 1 (2.6) | 0 |
| Cardiac failure | 1 (0.4) | 0 | 1 (2.6) | 0 |

**Supplementary Table S7.**

**Baseline characteristics and safety profile in patients receiving concomitant radiation therapy for metastases (N=31)**

| **Baseline characteristics** | **N=31** | |
| --- | --- | --- |
| Age |  | |
| Median (range), years | 64 (37–84) | |
| ≥75 years, n (%) | 5 (16.1) | |
| Sex^a^, n (%) |  | |
| Male | 25 (80.6) | |
| Female | 6 (19.4) | |
| Karnofsky score, n (%) |  | |
| ≤70 | 8 (26.6) | |
| 80–100 | 22 (73.3) | |
| Data missing | 1 (3.2) | |
| Number of prior therapies, n (%) |  | |
| 1 | 24 (77.4) | |
| 2 | 7 (22.6) | |
| Histology, n (%) |  | |
| Clear cell | 30 (96.8) | |
| Nephrectomy, n (%) |  | |
| Yes | 22 (71.0) | |
| No | 9 (29.0) | |
| Location of metastases treated, n (%) |  | |
| Bone | 20 (64.5) | |
| Brain | 5 (16.1) | |
| Lung | 4 (12.9) | |
| Adrenal gland | 1 (3.2) | |
| Kidney | 1 (3.2) | |
| Soft tissues | 1 (3.2) | |
| Metastatic lymph nodes | 1 (3.2) | |
| **Safety profile** | **Overall** | **Within 3 months of RT** |
| AEs, n (%) | 29 (90.6) | 16 (50.0) |
| SAEs | 24 (75.0) | 7 (21.9) |
| Treatment-related AEs, n (%) | 17 (53.1) | 7 (21.9) |
| Treatment-related SAEs, n (%) | 4 (12.5) | 7 (21.9) |
| Treatment-related SAEs leading to death, n | 0 | 0 |

AE, adverse event; RT, radiation therapy; SAE, serious adverse event.
^a^As determined by a set of biological attributes that are associated with physical and physiological features.
